# Identification of mIDH1 R132C/S280F Inhibitors from Natural Products by Integrated Molecular Docking, Pharmacophore Modeling and Molecular Dynamics Simulations

**DOI:** 10.3390/ph17030336

**Published:** 2024-03-05

**Authors:** Weitong Zhang, Hailong Bai, Yifan Wang, Xiaorui Wang, Ruyi Jin, Hui Guo, Huanling Lai, Yuping Tang, Yuwei Wang

**Affiliations:** 1 College of Pharmacy, Shaanxi University of Chinese Medicine, Shiji Ave., Xi’an-Xianyang New Ecomic Zone, Xianyang712046, China; 222031711625@email.sntcm.edu.cn (W.Z.); 223040011852@email.sntcm.edu.cn (H.B.); 222031712627@email.sntcm.edu.cn (Y.W.); jinruyi335588@163.com (R.J.); guohui@sntcm.edu.cn (H.G.); yupingtang@sntcm.edu.cn (Y.T.); 2 Dr. Neher’s Biophysics Laboratory for Innovative Drug Discovery, State Key Laboratory of Quality Research in Chinese Medicine, Macau Institute for Applied Research in Medicine and Health, Macau University of Science and Technology, Macao 999078, China; wangxr2018@lzu.edu.cn; 3 Guangzhou Laboratory, Guangzhou 510005, China; laihl910@126.com

**Keywords:** IDH1 R132C/S280F mutations, molecular docking, pharmacophore modeling, ADMET, molecular dynamics simulations

## Abstract

Mutant isocitrate dehydrogenase 1 (mIDH1) is a common driving factor in acute myeloid leukemia (AML), with the R132 mutation accounting for a high proportion. The U.S. Food and Drug Administration (FDA) approved Ivosidenib, a molecular entity that targets IDH1 with R132 mutations, as a promising therapeutic option for AML with mIDH1 in 2018. It was of concern that the occurrence of disease resistance or recurrence, attributed to the IDH1 R132C/S280F second site mutation, was observed in certain patients treated with Ivosidenib within the same year. Furthermore, it should be noted that most mIDH1 inhibitors demonstrated limited efficacy against mutations at this specific site. Therefore, there is an urgent need to investigate novel inhibitors targeting mIDH1 for combating resistance caused by IDH1 R132C/S280F mutations in AML. This study aimed to identify novel mIDH1 R132C/S280F inhibitors through an integrated strategy of combining virtual screening and dynamics simulations. First, 2000 hits were obtained through structure-based virtual screening of the COCONUT database, and hits with better scores than −10.67 kcal/mol were obtained through molecular docking. A total of 12 potential small molecule inhibitors were identified through pharmacophore modeling screening and Prime MM-GBSA. Dynamics simulations were used to study the binding modes between the positive drug and the first three hits and IDH1 carrying the R132C/S280F mutation. RMSD showed that the four dynamics simulation systems remained stable, and RMSF and Rg showed that the screened molecules have similar local flexibility and tightness to the positive drug. Finally, the lowest energy conformation, hydrogen bond analysis, and free energy decomposition results indicate that in the entire system the key residues LEU120, TRP124, TRP267, and VAL281 mainly contribute van der Waals forces to the interaction, while the key residues VAL276 and CYS379 mainly contribute electrostatic forces.

## 1. Introduction

The somatic mutations of IDH1 play a pivotal role in the initiation and maintenance of various types of cancers [1,2], such as AML [3,4,5], intrahepatic cholangiocarcinoma [6], grade II–III gliomas [7], and secondary glioblastomas [8]. IDH1 is a crucial metabolic enzyme that facilitates the oxidative decarboxylation of isocitrate to α-ketoglutarate (α-KG) in the tricarboxylic acid cycle (TCA), predominantly localized in the cytoplasm. Once mutation of IDH1 occurs, the conversion of α-KG to R-2-hydroxyglutaric (2-HG) acid catalyzed by mIDH1 [9]. The development of AML in patients with IDH1 mutations is often associated with abnormally elevated 2-HG levels [10], and accumulated 2-HG occupies the active site of α-KG-dependent dioxygenase and competitively inhibits its activity. The inhibition hinders the conversion of 5-methylcytosine to 5-hydroxycytosine, thereby impairing the process of DNA demethylation [11,12,13,14,15,16,17]. Simultaneously, it inhibits the expression and regulation of JmjC domain-containing histone demethylases in cell differentiation [18,19], resulting in DNA hypermethylation and epigenetic dysregulation. In summary, increased levels of 2-HG due to mutations promote the occurrence and development of AML.

The oral mIDH1 R132 inhibitor, Ivosidenib, was first approved by the FDA for the treatment of adult patients with AML carrying mIDH1 [20,21]. Ivosidenib treatment is both effective and safe, capable of reducing or even eliminating the variant allele frequency, thereby providing deep and long-lasting relief for some patients [22]. However, resistance was observed in some patients treated with Ivosidenib during the same year [23]. This resistance mechanism involves specific mutations in the IDH1, including secondary site mutations at R132C and S280F [24]. When patients developed IDH1 R132C/S280F mutations, the efficacy of Ivosidenib as an inhibitor against these variants was compromised, thereby significantly undermining its therapeutic potential for patients with AML [24,25,26]. Additionally, for patients who have undergone Ivosidenib treatment, the presence of mutations can result in treatment failure and increase the risk of disease recurrence, posing a significant threat to patient survival and quality of life. Consequently, there is an urgent need to explore novel therapeutic approaches targeting resistance mechanisms associated with mIDH1, which have great potential for therapeutic options in AML patients, especially approaches developed at secondary sites.

DS-1001b, a compound specifically targeting mIDH1 R132, has successfully progressed into phase 2 clinical trials and demonstrates the ability to reduce 2-HG levels in oral molecule inhibitor-induced tumors [27]. The tolerability and permeability of the blood–brain barrier were found to be excellent for DS-1001b, as demonstrated by previous studies [28,29]. Reinbold et al. conducted biochemical and cytologic investigations on DS-1001b and other agents in phase 2 clinical trials targeting the second site of mIDH1 R132C/S280F. The findings demonstrated that treatment with DS-1001b significantly reduced 2-HG levels in R132C/S280F cells and exhibited a robust binding affinity to mIDH1 of R132C/S280F. These results suggested that DS-1001b showed potential for overcoming S280F mediated drug resistance in AML patients [30]. However, since DS-1001b is in the second phase of clinical trials and mainly targets the R132 mutation, there has been no clinical trial on the target of the second site mutation. Therefore, there is an urgent need to develop an mIDH1 that can inhibit the second site mutation of inhibitors.

Nowadays, structure-based virtual screening has emerged as a rapid and cost-effective method for identifying potential lead compounds. Unlike high-throughput screening assays, structure-based virtual screening enables the prediction of compound affinity towards specific binding sites [31,32,33,34]. For example, our group previously used a docking-based virtual screening strategy to screen 57 structurally different IDH1 R132H inhibitors and identified 10 highly active compounds through experimental testing [31]. In addition, other international groups have also used virtual screening technology to discover mIDH1 small molecule inhibitors with diverse structures [35,36]. In addition to synthetic small molecule compounds, natural products are also an important source of potential inhibitors of mIDH1. Zheng et al. identified three natural steroids from Ganoderma sinense, a unique medicinal edible fungus native to China [37], while Zhou et al. discovered Styraxlignolide F and Tremulacin as potential mIDH1 inhibitors through virtual screening technology [38]. All compounds showed high specificity for the target enzyme and demonstrated low toxicity in experimental studies.

Natural products refer to bioactive small molecules synthesized within living organisms, exhibiting diverse biological activities that render them promising candidates for applications in pharmacology and various industries [39,40]. According to the statistics and categorization of drugs approved by the FDA over the past 39 years conducted in 2020, as presented by David J. Newman and Gordon M. Cragg, it was discovered that more than 45% of pharmaceuticals were derived from natural products and their derivatives [41], particularly focusing on antibacterial and antitumor agents. The COCONUT database is a compilation of 53 different databases and literature, encompassing 426,916 natural products lacking stereochemical structures and 746,626 natural products with stereochemical structures. This comprehensive dataset comprises both elucidated and predicted natural products from various open sources [42]. Therefore, virtual screening technology from the COCONUT database can be used to discover potential inhibitors targeting mIDH1 R132C/S280F.

In this study, initial virtual screening was conducted using Glide docking and the pharmacophore model to identify inhibitors targeting mIDH1 from the COCONUT database of 407,270 compounds. Subsequently, dynamics simulations [43,44] and binding free energy calculations [45] were performed to further investigate the detailed dynamic mechanisms between inhibitors (positive drugs and selected compounds) and crystal structures of IDH1 with R132C/S280F mutations.

## 2. Results and Discussion

### 2.1. Virtual Screening of Natural Compounds for mIDH1 Inhibitor

**Figure 1:**
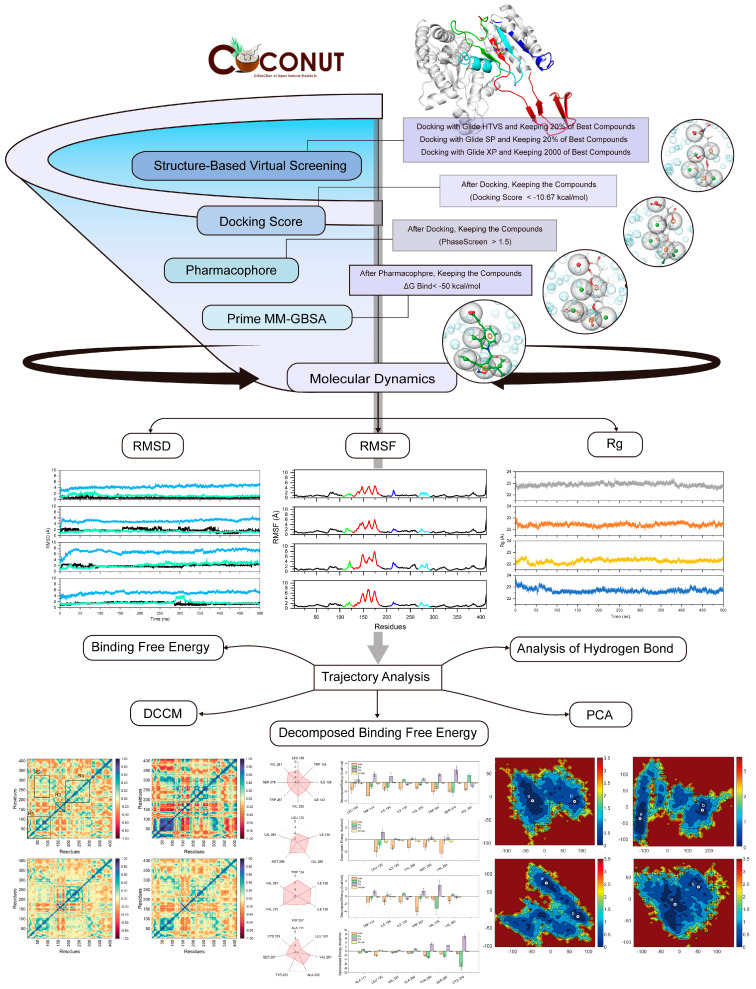
The flowchart of virtual screening and dynamics simulations.

**Figure 2:**
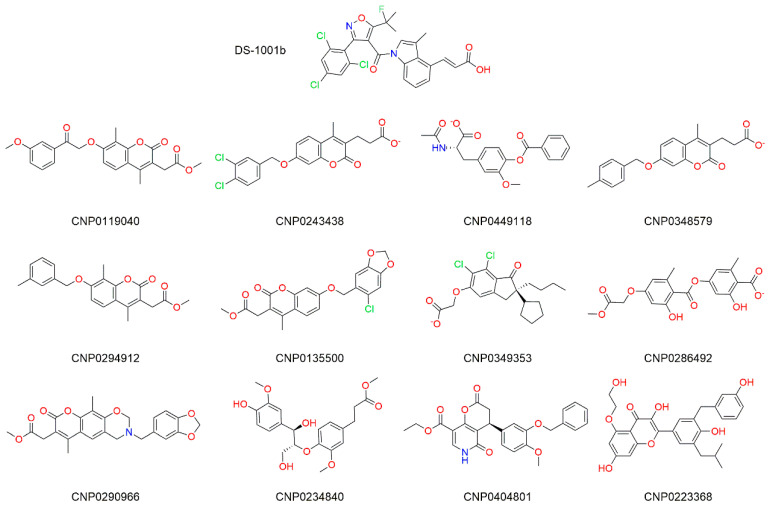
Chemical structures of the 12 compounds identified by structure-based virtual screening and pharmacophore analysis.

To identify mIDH1 R132C/S280F inhibitors, we first employed virtual screening to discover the targeted compounds with the best docking score. The workflow of virtual screening is depicted in Figure 1. Following a three-stage screening process involving HTVS, SP, and XP, a total of 2000 compounds were identified, exhibiting the highest docking score obtained from Glide screening. Among them, 280 molecules had docking scores lower than −10.67 kcal/mol and were selected for further analysis. After conducting pharmacophore screening, this number was eventually reduced to 28 molecules. The Prime MM-GBSA analysis was performed on these 28 molecules, with ΔG Bind being chosen as the parameter. Consequently, a total of 12 molecules displaying an affinity of less than −50 kcal/mol were identified as potential inhibitors (Table 1 and Figure 2). The three molecules that exhibited optimal performance in Prime MM-GBSA were chosen as inhibitors for subsequent investigations. As shown in Figure 2, the chemical structure of the compounds has a similar core structure to coumarins and flavonoids. However, the compounds were clustered with coumarin and flavonoids through fingerprinting (Figure S1 in Supplementary Materials), and the results showed that the similarity was not high, indicating that the structure was diverse.

**Figure 3:**
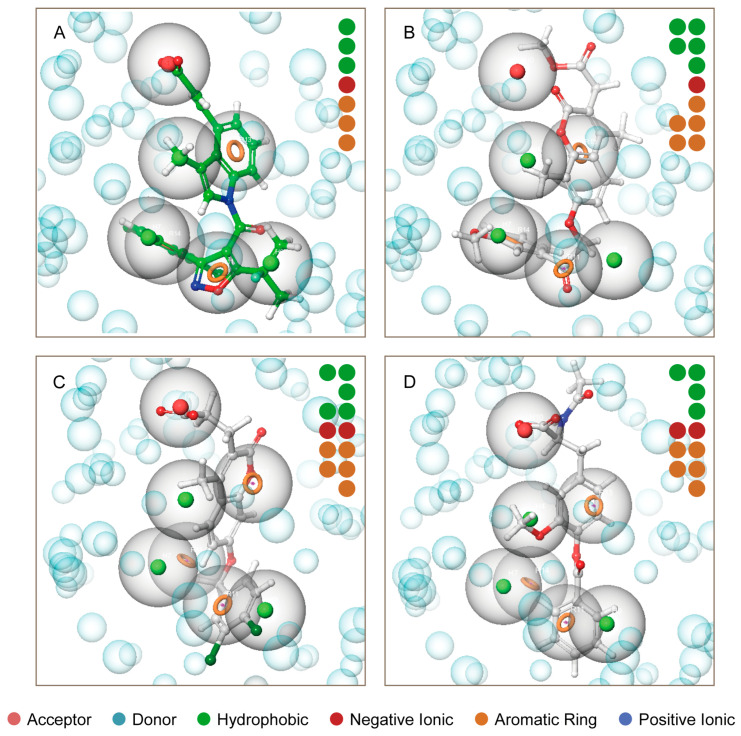
Pharmacophore model of the three molecules and DS-1001b. The pharmacophore models of DS-1001b, CNP0119040, CNP0243438, and CNP0449118 are depicted in panel (**A**–**D**) respectively. The pharmacophore features include acceptor (Color: Brown), donor (Color: Sky blue), hydrophobic (Color: Forest green), negative ionic (Color: Firebrick red), aromatic ring (Color: Orange), and positive ionic (Color: Deep blue).

The presence of seven pharmacophores can be observed in DS-1001b (Figure 3A). The carboxyl group at the terminal end of the molecule exhibits negative ionic properties, while benzene and trichlorophenyl in the indole ring contribute to its aromaticity. Furthermore, the three carbon atoms display hydrophobic characteristics. The two methyl groups on CNP0119040 (Figure 3B) overlap with the hydrophobic region of DS-1001b, while the benzene rings on methoxyphenyl and chromene align with two aromatic ring pharmacophores. In CNP0243438 (Figure 3C) and CNP0449118 (Figure 3D), the terminal carboxyl group coincides with a negative ionic pharmacophore, while the two benzene rings coincide with an aromatic ring pharmacophore. The two hydrophobic groups in CNP0243438 correspond to a methyl group and two chlorine atoms on chromene, respectively. Finally, in CNP0449118, the carbon atom on the methyl ether group corresponds to a hydrophobic pharmacophore.

### 2.2. ADME Prediction

The Lipinski five rules were proposed by American medicinal chemist Christopher A. Lipinski in 1997 for the evaluation of molecular drug candidates, which encompass the following criteria: (1) Molecular mass (MW) < 500; (2) lipid solubility (QplogPo/w) < 5; (3) the number of hydrogen bond donors (HB) < 5; (4) the number of hydrogen bond acceptors (Accept HB) < 10. The information presented in Table 2 demonstrates that the values of QPlogPC16 range from 11.65 to 17.06, while the range for QPlogPoct is from 16.94 to 28.20. Additionally, QplogPw ranges from 7.37 to 17.66, and QPlogS ranges from −3.33 to −7.55. The CIQPlogS values fall within the range of −3.77 to −8.73, whereas the range for QplogHERG is between −2.69 and −6.63. The human oral absorption values are in the range of 1 to 3, with a percentage ranging from 59.66% to 100%. The ADME results demonstrated that the selected compounds exhibited adequate oral absorption and possessed suitable solubility and absorption properties in accordance with drug-like principles. The QPPCaco values of all compounds, except CNP0286492, exhibited satisfactory results. The range of QPlogBB was from −2.87 to −0.36, and the majority of compounds achieved good scores in terms of QPPMDCK, with the exception of CNP0286492. Human intestinal absorption plays a crucial role in determining drug bioavailability, while Caco-2 and MDCK cell lines are widely utilized in vitro models for evaluating intestinal drug absorption and blood–brain barrier permeability, respectively. The solubility and bioavailability parameters of these molecules, including QPlogPC16, QPlogPoct, QplogPw, QplogPo/w, CIQPlogS, Qplog HERG, QPPCaco, QPlogBB, QPPMDCK, and QPlogKp all exhibit favorable characteristics for human absorption. The bioactivity radar, BOILED-Egg plot, druglikeness and PAINS of hits were obtained through SwissADME analysis (Table S1, Figure S2 in Supplementary Materials). In the Figure S2, CNP0349353 and CNP0294912 showed better blood–brain barrier permeability (BBB), except DS-1001b; CNP0223368 and CNP0286492 demonstrated better human intestinal absorption (HIA), except CNP0404801; CNP0290966 and CNP0234840 displayed low P-glycoprotein permeability (PGP). Among these hits, CNP0349353 and CNP0294912 showed appropriate BBB permeability, excellent HIA, and low PGP permeability, and may be potential drug candidate compounds. None of the hits were identified as PAINS in the current PAINS screening method, and all of them exhibited good druglikeness.

### 2.3. The Stability of the mIDH1 Inhibitor System

**Figure 4:**
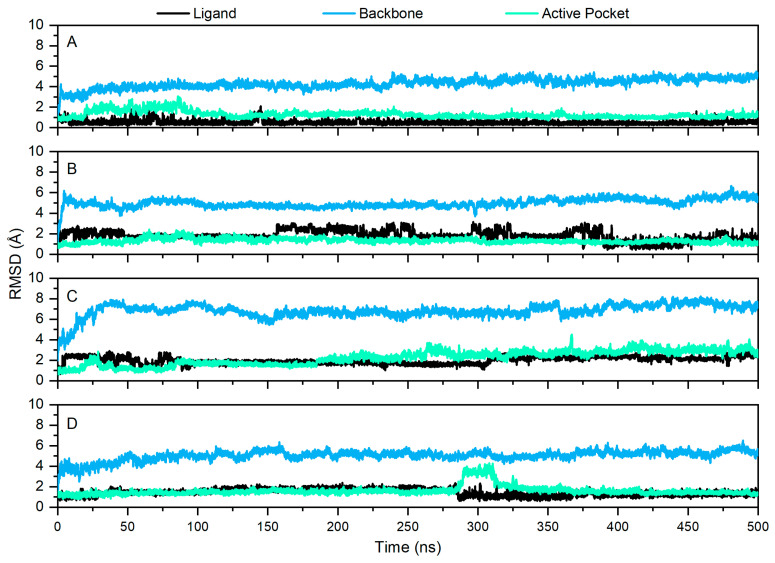
Fluctuation of RMSD values for DS-1001b and three molecules during 500 ns MD simulations. The fluctuation of RMSD values of DS-1001b, CNP0119040, CNP0243438, and CNP0449118 are depicted in panels (**A**–**D**), respectively. The black, blue, and cyan fluctuations corresponded to the ligand, backbone, and active pocket, respectively.

To further investigate the molecular interaction with mIDH1, MD simulations were performed using AMBER 18 on DS-1001b, CNP0119040, CNP0243438, and CNP0449118. The RMSD was widely employed for evaluating the stability and dynamics of the overall molecular structure. Simulations were performed for a duration of 500 ns, during which the stabilities of mIDH1 backbone, active pocket, and ligand were assessed by calculating their RMSD values. As depicted in Figure 4, it can be observed that RMSD fluctuations remained within a range of 2 Å after 400 ns as detected. DS-1001b (Figure 4A) exhibited a lower RMSD amplitude compared to other identified inhibitors, indicating its superior stability in terms of active pocket dynamics. In contrast, CNP0119040 (Figure 4B) and CNP0449118 (Figure 4D) demonstrated more pronounced stability in their respective active pockets, while CNP0243438 (Figure 4C) displayed enhanced overall system stability.

**Figure 5:**
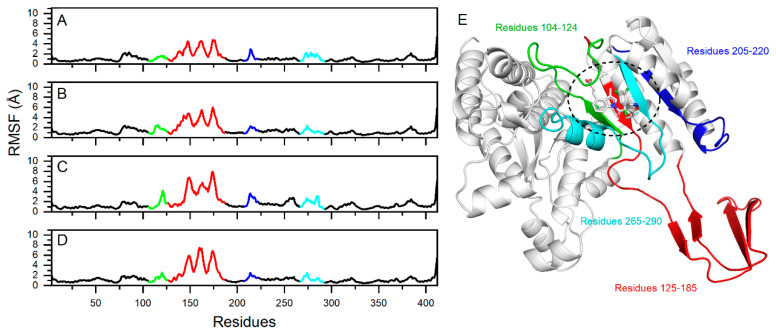
RMSF values of mIDH1 residue backbone and visualization. The fluctuation of RMSF values of DS-1001b, CNP0119040, CNP0243438, and CNP0449118 are depicted in panels (**A**–**D**), respectively. The RMSF fluctuations on the crystal structure are depicted in (**E**). Residues 104–124 are illustrated in green, while residues 205–220 are shown in blue. The positions of residues 265 to 290 are indicated by cyan, and red indicates residues 125 to 185.

The RMSF analysis is a widely employed method for assessing the local flexibility and fluctuations of protein molecules. Simulations results indicate that the RMSF values of these four systems exhibit similar trends, thereby demonstrating the dynamic impact of inhibitors on protein interiors. The region spanning residues 125 to 185 (Figure 5E) displayed significant flexibility across all systems, with the IDH1 of R132C/S280F mutation bound to DS-1001b (Figure 5A–D) exhibiting the lowest RMSF value among the complexes. In Figure 5A–D, the regions 104–124, 265–290, and 205–220 show differences in RMSF values (>2 Å). The regions were primarily localized in close proximity to the binding site, as illustrated in Figure 5E. Generally, higher flexibility is associated with a decrease in compactness and intramolecular hydrogen bonds, potentially impacting the distance between crucial residues.

**Figure 6:**
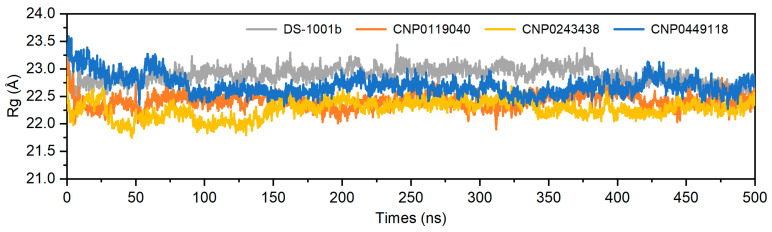
The Rg values of the DS-1001b, CNP0119040, CNP0243438, and CNP0449118 (frame interval = 10).

The radius of gyration (Rg) is commonly employed for assessing the overall compactness and structural evolution. As depicted in Figure 6, the Rg values of these four complexes remained stable throughout 500 ns dynamics simulations, with their structures exhibited a level of compactness comparable to that of the positive drug DS-1001b. The analysis demonstrates that the stability of each system was maintained throughout the simulations.

### 2.4. Dynamic Cross-Correlation Maps and Free Energy Landscapes

**Figure 7:**
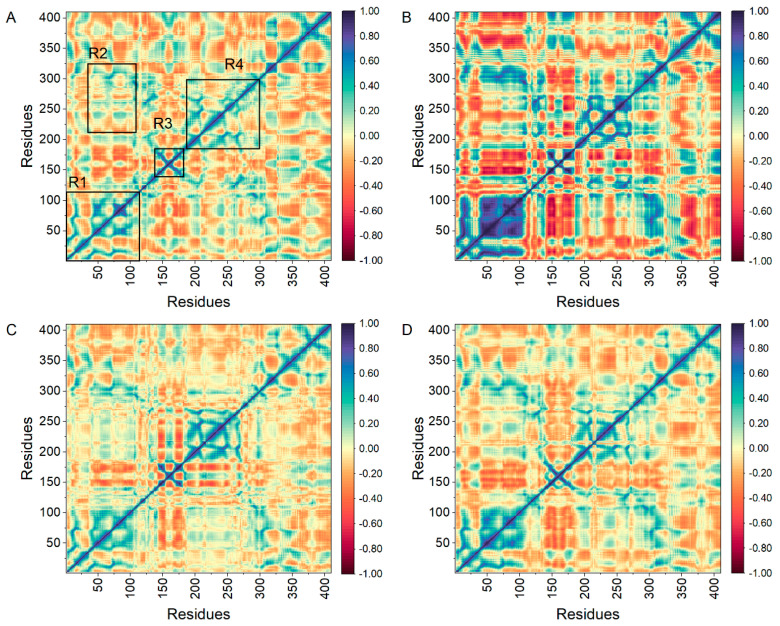
DCCM of the three molecules and DS-1001b. The DCCM of DS-1001b, CNP0119040, CNP0243438, and CNP0449118 are depicted in panels (**A**–**D**) respectively.

The calculation of DCCMs was performed using the C-atom coordinates extracted from the MD trajectories to study the intrinsic dynamic association and synergy of inhibitors with mIDH1. Figure 7 depicts the correlated movements between residues in the four systems, with blue regions indicating strong positive correlations among residue movement and red regions representing robust inverse correlations among residue movement. The diagonal component primarily characterizes the positive correlation motion with respect to their own residues, while the off-diagonal region mainly reflects the anti-correlation motion or synergy between residues. The movement patterns of residues are found to be similar in the four systems, as depicted in Figure 7. Notably, significant alterations in these patterns are observed within the regions highlighted by black boxes. The diagonal elements of the R1 region in mIDH1-DS-1001b (Figure 7A) exhibit a robust positive correlation among residues. Residues within the R3 and R4 regions predominantly display positively correlated motions. In contrast, the R2 region demonstrates pronounced anticorrelation motions along with subtle positive correlations between residues. Compared to DS-1001b, the binding of CNP0119040 (Figure 7B) significantly enhanced positive correlation motion in the R1 region, while CNP0119040, CNP0243438 (Figure 7C), and CNP0449118 (Figure 7D) notably increased positive correlation motion in R3. The observed significant changes in movement patterns within the R3 corresponded to previous RMSF findings. Hence, distinct substitutions at the same position can induce variations in mDIH1 internal dynamics.

The energy landscape serves as a valuable tool for elucidating the energetic underpinnings of protein conformational changes, with each distinct energy depression in the figure representing a unique lowest energy conformation. The motion information within the system is obtained by performing principal component analysis, which is a method employed to comprehend the dynamics of molecular trajectories. The eigenvalues and focus matrices were obtained through orthogonal coordinate transformations. The principal components were derived from the eigenvectors and eigenvalues. The motion of the system in two dimensions is reflected by the horizontal and vertical coordinates, respectively.

**Figure 8:**
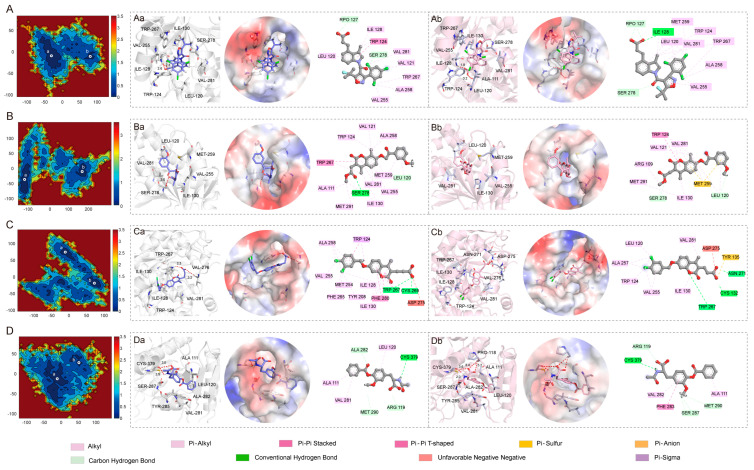
Free energy landscapes of the DS-1001b (**A**), CNP0119040 (**B**), CNP0243438 (**C**), and CNP0449118 (**D**). In the figure, (**Aa,Ab,Ba,Bb,Ca,Cb,Da,Db**) correspond to the lowest conformations a and b of A, B, C and D in the free energy landscape, respectively.

The free energy landscapes, 3D binding models, protein contact potential, and 2D binding models are presented in Figure 8. In the free energy landscape, the mIDH1 (Figure 8A–D) systems have achieved the two lowest energy conformations. The conformational transitions within each complex are delineated by a subspace, indicating that these small molecule inhibitors bind to the protein through different binding modes, resulting in minimal binding effects. Based on these principal components, representative conformations during the simulations were extracted. The 3D and 2D binding modes between DS-1001b and mIDH1 (Figure 8A) reveal that the carbonyl group in the molecule forms hydrogen bonds with SER 278, while carboxyl groups form hydrogen bonds with RPO127. Additionally, indole rings exhibit pi-alkyl interactions with LEU120 and ILE128, and TRP267, ALA258, and VAL255 engage in pi-alkyl interactions with trichlorophenone rings. In the binding mode of CNP0119040 and mIDH1 (Figure 8B), there is a significant conformational change in the position of the carbon–oxygen bond, leading to substantial alterations in the docking posture of small molecules. Specifically, anisole forms hydrogen bonds with LEU120, MET259 interacts with anisole, and benzofuran rings form pi-alkyl interactions with VAL281 and VAL255 in Figure 8(Ba). However, in Figure 8(Bb), due to rotation of the carbon–oxygen bond, the benzofuran ring forms a pi-alkyl interaction with TRP124 and VAL281 instead. In the binding mode between CNP0243438 and mIDH1 (Figure 8C), TPR267 forms intermolecular hydrogen bonds with the carboxyl group oxygen, while benzofuran interacts through pi-alkyl interactions with ILE130. In Figure 8(Ca), VAL276 forms hydrogen bonds with the carboxylic acid oxygen, whereas dichlorobenzene rings interact via pi-alkyl interactions with ALA258, VAL255, MET254, TYR208, and PHE265. In Figure 8(Cb), the carboxylic acid oxygen forms hydrogen bonds with ASN271, CYS132 and TRP267 form hydrogen bonds with benzofuran oxygen, dichlorobenphenyl ring interacts via pi-alkyl interactions with LEU120, ALA257, TRP124, and VAL255. Although the rotation of carbon–hydrogen bonds causes a shift in the position loop of dichlorobenzene rings in the diagram, it can still be observed that small molecules interacted with TRP267, ASP275 ILE130, and VAL255. In the binding mode of CNP0449118 and mIDH1 (Figure 8D), ALA111 engages in pi-alkyl interactions with benzene rings, and CYS379 establishes hydrogen bonds with nitrogen atoms on acetamide. In Figure 8(Da), ALA282 forms hydrogen bonds with oxygen atoms in ester groups, VAL281 engages in pi-alkyl interactions with benzene rings, and ALA282 forms hydrogen bonds with oxygen atoms in the ester groups. In Figure 8(Db), SER287 and MET290 form a C-H bond with the end-group carbon of the ether bond, and ligand 283 forms a pi-pi T-shaped bond with the benzene ring. Consistent with the decomposition free energy structure, LEU120, TRP124, and TRP267 predominantly contribute to van der Waals interactions, while VAL281 and VAL276 mainly provide electrostatic energy. Additionally, CYS379 is primarily responsible for the electrostatic energy contribution. The protein contact potential of Figure 8 is a qualitative electrostatic representation generated by PyMOL. The active pocket contains a combination of different postures exhibited by all the small molecules. In particular, CNP0119040 adopts a relatively folded posture within the active pocket, with Figure 8(Bb) showing an even more pronounced folding compared to Figure 8(Ba). The active pocket exhibits a relatively stretched conformation for CNP0243438. Upon comparing the two lowest energy conformations, it is evident that the o-dichlorobenzene ring in the molecule undergoes stretching in opposite directions (Figure 8(Ca,Cb)). This phenomenon may be attributed to the free rotation of the carbon group connected to the o-dichlorobenzene ring, which prevents stable interaction with the active pocket. The binding mode of NP0119040, CNP0243438, and CNP0449118 to mIDH1 was found to have a lower degree of interaction with active sites compared to DS-1001b. This observation is consistent with the results obtained from the analysis of their binding affinities.

### 2.5. Analysis of Hydrogen Bond

Hydrogen bonding plays a crucial role as one of the most significant non-covalent interactions in the binding of molecules to mIDH1. The hydrogen bond changes between individual residues and inhibitors were monitored over 50–500 ns to investigate the interaction between the molecules and mIDH1. The amino acids listed in Table 3 exhibit hydrogen bond occupancy exceeding 1%. The findings demonstrate that compound DS-1001b, along with ILE128 and ALA111, display occupancies of 30.30% and 2.93%, respectively. Compounds CNP0119040 and LEU120 exhibit an occupancy of 14.08%. Additionally, CNP0243438 and TRP267 account for occupancies of 2.22%, while CNP0449118 and CYS379 contribute to an occupancy of 16.53%. The compounds of NP0119040, CNP0243438, and CNP0449118 exhibit a reduced proportion of hydrogen bonding with mIDH1 in comparison to DS-1001b, suggesting that electrostatic forces play a crucial role in maintaining the stability of small molecule binding to mIDH1.

### 2.6. Analysis of Binding Free Energy

The binding free energy was utilized as a reference standard for evaluating molecular activity. It is widely acknowledged that a lower binding value indicates greater stability of the protein–small molecule complex formed. To evaluate the binding affinity of each complex, the MM-GBSA method was employed to calculate the binding free energy (ΔGbind), aiming to investigate the structural basis and crucial residues involved in DS-1001b, CNP0119040, CNP0243438, and CNP0449118:

$$\Delta G_{\mathrm{gas}}=\Delta E_{\mathrm{ele}}+\Delta E_{\mathrm{vdw}}$$

$$\Delta G_{\mathrm{sol}}=\Delta G_{\mathrm{GB}}+\Delta G_{\mathrm{GBSUR}}$$

$$\Delta G_{\mathrm{bind}}=\Delta G_{\mathrm{gas}}+\Delta G_{\mathrm{sol}}$$

The binding free energies of DS-1001b, CNP0119040, CNP0243438, and CNP0449118 were −36.951 kcal/mol, −28.74 kcal/mol, −31.32 kcal/mol, and −24.75 kcal/mol, respectively (as shown in Table 4). Among these values, the electrostatic energy (ΔEele) was significantly lower than other energy terms except for CNP0119040 (−57.11 kcal/mol, −7.55 kcal/mol, −74.58 kcal/mol, and −81.80 kcal/mol respectively). This observation suggests that hydrophobic interactions play a major role in the ligand binding process. The van der Waals energy (ΔEvdw) values were −49.51 kcal/mol, −39.97 kcal/mol, −43.70 kcal/mol, and −40.62 kcal/mol, respectively, indicating the positive role of van der Waals interactions in ligand binding. It is noteworthy that the polar contribution ΔG_GB_ negatively impacts ligand binding due to the involvement of numerous solvent molecules interacting with functional groups of the ligands, thereby increasing the entropy contribution and exacerbating the adverse effects of polarity. The larger value of ΔG_GB_ for CNP0449118 compared to other compounds in Table 4 indicates a relatively weaker binding affinity. Furthermore, Figure 8 reveals an obvious shift in the position of CNP0449118 when compared to DS-1001b, which may be attributed to local structural changes occurring in small and medium molecules during dynamics simulations, leading to an overall increase in free energy alteration.

**Figure 9:**
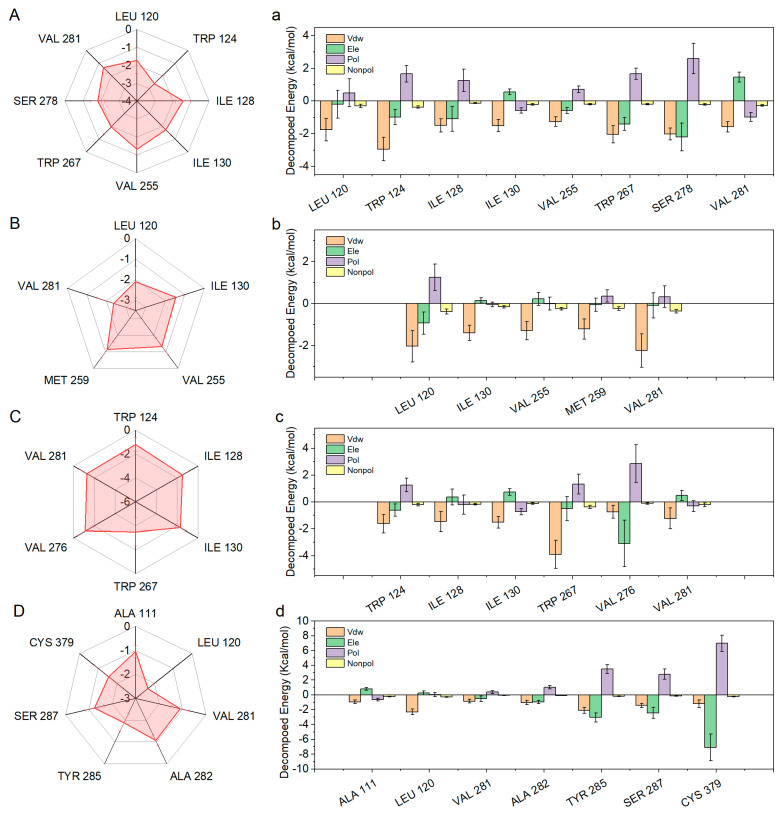
Decomposed binding energy of four inhibitors. The DCCM of DS-1001b, CNP0119040, CNP0243438, and CNP0449118 are depicted in panels (**A** (**a**), **B** (**b**), **C** (**c**), and **D** (**d**)) respectively.

The interaction energies of DS-1001b, CNP0119040, CNP0243438, and CNP0449118 were further analyzed to gain a deeper understanding of their interaction mechanism. Specifically, residue interaction energies exceeding −1.0 kcal/mol were decomposed into electrostatic interactions, van der Waals interactions, polar solvation free energy, and nonpolar solvation free energy as illustrated in Figure 9. The mIDH1-DS1001b system demonstrates key residues, specifically LEU120, TRP124, ILE128, ILE130, VAL255, TRP267, SER278, and VAL281. Van der Waals interactions play a significant role in the process of ligand binding. The polar effect has a negative impact on ligand binding. LEU120 forms a pi-alkyl interaction with the indole ring while TRP124 engages in p-p stacking with the five-membered ring on the indole moiety. The mIDH1-CNP0119040 (Figure 9B) system exhibits a significant interaction energy of more than 1.0 kcal/mol with the molecule, primarily attributed to van der Waals interactions involving five specific residues: LEU120, ILE130, VAL255, MET257, and VAL281. The TRP267 residue was p-p conjugated to the benzopyran loop on CNP0119040 (Figure 8B). In the mIDH1-CNP0243438 (Figure 9C) system, VAL276 primarily interacts with electrostatic forces and forms a hydrogen bond interaction with the carboxyl group at the C-terminus of CNP0243438 (Figure 8C), which aligns well with the principal component analysis results. The interaction energies of TRP124, ILE128, ILE130, TRP267, and VAL281 with molecules primarily arise from van der Waals interactions. In the mIDH1-CNP0449118 (Figure 9D) system, the interaction force between CYS379 and molecules is predominantly derived from electrostatic energy through hydrogen bond formation with N-H bonds on small molecules (Figure 8D), consistent with the principal component analysis results.

## 3. Materials and Methods

### 3.1. Preparation of Receptor and Ligands

The X-ray crystal structure of the Ivosidenib-resistant IDH1 variant R132C/S280F in complex with NADPH and inhibitor DS-1001b (PDB ID: 7PJN; Resolution: 2.45 Å) [30] was obtained from the Protein Data Bank [46] (PDB: https://www.rcsb.org/, accessed on 28 November 2022). The protein preparation wizard panel in Schrödinger 2015 [47] (Release 2015, Schrödinger, LLC, New York, NY, USA)was utilized to incorporate hydrogen atoms, remove all water molecules, assign charges and protonation states at pH 7.0, and optimize the structure using the OPLS-2005 force field [48]. The receptor grid in Schrödinger 2015 was generated using the OPLS-2005 force field through Glide receptor grid generation. The DS-1001b was designated as the central reference point of the grid, and the grid box was defined to docking ligands that are comparable in size to the native ligand.

The COCONUT (version 2022, Collection of Open Natural ProdUcTs Online, accessed on 8 November 2022) database was utilized as the screening source, containing approximately 407,270 molecules. The chemical structures were prepared using the LigPrep Panel in Schrödinger 2015 with the OPLS-2005 force field. The possible states of molecules were generated using Epik [49] at pH 7.0 ± 2.0, retained specified chiralities, followed by tautomer generation and the generation of up to 8 low-energy conformations per ligand.

### 3.2. Structure-Based Virtual Screening

The generated grid and prepared ligands were subjected to structure-based virtual screening using the virtual screening workflow of Glide [50]. The prepared ligands underwent prefiltration using QikProp to obtain the required properties, followed by application of Lipinski’s rule of five [51]. Ligands containing reactive functional groups were subsequently excluded.

The Glide screening consists of three docking stages. The initial stage is dedicated to HTVS docking, which enables rapid high-throughput screening. The molecules that pass this stage proceed to the subsequent stage, where SP docking is performed. At each stage, only the top 20% ranked molecules are retained for further evaluation as potential inhibitors. The surviving molecules from this selection process then advance to the third and final stage, where XP docking is conducted with a retention of 2000 molecules.

### 3.3. Pharmacophore-Based Virtual Screening

In this study, the phase module of Schrödinger 2015 was employed to generate an e-pharmacophore [52] hypothesis. The hypothesis was formulated based on the complementarity between the receptor and ligand features, utilizing the crystal structure of mIDH1 complexed with DS-1001b (PDB ID: 7PJN). The ligands were screened from the database using a threshold of phase screen score >1.5 to assess how well the ligands fit the respective hypothesized pharmacophore characteristics for which they were screened. The phase module provides 6 hypotheses, such as acceptor (A), donor (D), hydrophobic (H), negative ionic (N), positive ionic (P), aromatic ring (R), which were used to interpret ligand–receptor interactions.

### 3.4. ADME Prediction and Prime MM-GBSA

The ADME [53] (adsorption, distribution, metabolism, and excretion) properties of the compounds are determined using the QikProp module, a rapid and precise ADME prediction program that calculates physically significant descriptors and pharmaceutically relevant properties of organic molecules. It evaluates drug-likeness and pharmaceutical factors for all hits. Bioavailability, solubility, druglikeness and pan-assay interference compounds [54,55] (PAINS) of hits were calculated using SwissADME [56] (www.swissadme.ch, accessed on 20 February 2024) servers.

The Prime MM-GBSA (molecular mechanics with Generalized Born surface area) method in Maestro was employed to calculate the binding free energy of potential inhibitors to the mIDH1 R132C/S280F crystal structure, by calculating the fingerprints through canvas [57] and comparing their similarity.

### 3.5. Molecular Dynamics Simulations

To investigate the binding mode of inhibitors to mIDH1, molecular dynamics (MD) simulations were performed on the complex formed by mIDH1 and DS-1001b, as well as the representative active compounds identified. The initial structure of the mIDH1-DS-1001b complex was obtained from the PDB. The bond charge corrections (BCCs) [58] were utilized to fit the partial charges for the inhibitors. The general AMBER [59] force field (GAFF) [60] was employed for parameterizing the compounds, while the AMBER ff14SB force field [61] was used for the mIDH1 structure. The complex was solvated in TIP3PBOX at a distance of 12 Å from the boundary. After adding chloride and sodium ions to neutralize each system, the steepest descent method followed by the conjugate-gradient method were employed to minimize the system every 2500 steps. Subsequently, the systems were heated in the NVT ensemble from 0 to 310 K over a period of 500 ns, with restraints applied on backbone atoms. The restraint force was gradually reduced from 10 to 0.1 kcal/(mol·Å^2^) within 0.9 ns. The system was subjected to 500 ns molecular dynamics simulations at 310 K under 1 atmospheric pressure in an NPT ensemble, without any restraints. Trajectory analyses were conducted using the Cpptraj module in AMBER 18.

### 3.6. Calculation of Binding Free Energy

After MD simulations, trajectory analysis was performed using the Cpptraj module of AMBER 18. First, the root mean square deviation (RMSD) and root mean square fluctuation (RMSF) were calculated based on the MD trajectory. Subsequently, the last 5000 frames out of a total of 25,000 frames were selected to calculate the binding free energy and the free energy of decomposition using the molecular mechanics/Generalized Born surface area (MM/GBSA) [62] methodology. The following formula is commonly employed in MM-GBSA calculations:

$$\Delta G_{\mathrm{bind}}=G_{\mathrm{complex}}-\left(G_{\mathrm{protein}}+G_{\mathrm{ligand}}\right)$$

where the energy ΔG is expressed as follows:

$$\Delta G=\Delta E_{\mathrm{ele}}+\Delta E_{\mathrm{vdw}}+\Delta G_{\mathrm{GB}}+\Delta G_{\mathrm{GBSUR}}-T\Delta S$$

in which the first two components represent the electrostatic and van der Waals interactions in the gas phase, respectively. The third term corresponds to the electrostatic polar solvation free energy, which can be determined using the Generalized Born (GB) equation. The fourth term represents the nonpolar solvation free energy, while neglecting any entropy changes in conformation. Key residues involved in the binding process were identified by decomposing the binding free energy into individual residue contributions.

### 3.7. The Computation of DCCM and PCA

The Cpptraj module [63] in Amber 18 was employed for the computation of dynamic cross-correlation maps [64,65] (DCCM) and principal component analysis (PCA). DCCM was utilized to analyze the internal dynamics in biomolecules during molecular simulations by calculating correlation coefficients between different regions within residues based on trajectory data, followed by plotting correlation matrices using Origin 2021. PCA was performed by diagonalizing the position covariance matrix, constructed from the retained Cα atomic coordinates in the MD trajectory. The feature vectors generated through PCA represent correlated displacements of atom groups in multidimensional space, with eigenvalues indicating motion magnitude along each vector. The PCA analysis and mapping were conducted using MATLAB, while the visualization of the lowest energy conformations was achieved through PyMol 2.6 [66], where the electrostatic potential was obtained in vacuum. The 2D structure was completed with Discovery Studio 2020 [67] (Release 2020, Accelrys Software Inc, San Diego, CA, USA).

## 4. Conclusions

This study aimed to obtain inhibitors of the IDH1 R132C/S280F mutation by screening a natural product database. Virtual screening was employed to identify potential compounds and further investigated through dynamics simulations. Interestingly, despite the similar pharmacophore between the hits and DS-1001b, along with a comparable trend in RMSD, Rg, and binding affinity measurements, the hit compound still has lower binding affinity than DS-1001b. The unique structure of the natural product may have limitations compared to DS-1001b, which may be the reason why the hit compound binds incompletely to IDH1 of the second site mutation. The hits were further analyzed by dynamics simulations to obtain the interaction between the hits and the IDH1 of second site mutations. Among them, the key residues LEU120, TRP124, TRP267, and VAL281 were identified as the main contributors to van der Waals energy, while VAL276 and CYS379 were found to be a major source of electrostatic energy. This study establishes a theoretical foundation for the development of inhibitors that can overcome the R132C/S280F mutation-induced drug resistance.

## Figures and Tables

**Table 1:** The MW, docking score, phase screen score and Prime MM-GBSA energy of the top 12 compounds screened.

ID	MW	SP Docking Score (kcal/mol)	XP Docking Score (kcal/mol)	Phase Screen Score	Prime MM-GBSA (kcal/mol)
DS1001b	535.79	−10.67	−14.09	2.61	−84.90
CNP0119040	410.42	−8.60	−10.75	1.53	−65.57
CNP0243438	407.25	−8.84	−11.83	1.54	−60.72
CNP0449118	357.36	−9.61	−11.34	1.51	−59.10
CNP0348579	352.39	−8.72	−11.65	1.50	−57.48
CNP0294912	366.41	−9.89	−10.72	1.60	−54.00
CNP0135500	416.81	−8.68	−10.68	1.62	−53.26
CNP0349353	399.31	−8.79	−11.24	1.60	−52.91
CNP0286492	390.35	−9.57	−11.27	1.51	−52.79
CNP0290966	437.45	−10.03	−11.64	1.50	−51.28
CNP0234840	406.43	−6.72	−11.21	1.63	−51.13
CNP0404801	449.46	−9.09	−11.66	1.66	−50.77
CNP0223368	492.52	−9.49	−11.08	1.56	−50.47

**Table 2:** The Lipinski’s rule of five and ADME prediction.

ID	^a^ CNS	^b^ DonorHB	^c^ AccptHB	^d^ QplogPo/w	^e^ QPlogPC16	^f^ QPlogPoct	^g^ QplogPw	^h^ QPlogS	^i^ CIQPlogS	^j^ Qplog HERG	^k^ QPPCaco	^l^ QPlogBB	^m^ QPPMDCK	^n^ QPlogKp	# Metab	^o^ Qplog Khsa	^p^ Human Oral Absorption	^q^ Percent Human Oral Absorption
DS1001b	−1	1	6.50	5.82	15.14	22.340	10.80	−7.55	−8.73	−3.76	79.38	−0.91	394.98	−3.25	1	0.82	1	69.09
CNP0119040	−2	0	8.00	3.09	12.75	18.87	10.13	−4.69	−5.04	−5.88	424.82	−1.40	196.10	−2.81	6	−0.05	3	92.10
CNP0243438	−2	1	5.25	4.63	13.06	19.12	9.41	−6.21	−6.36	−4.19	84.18	−1.12	222.44	−2.95	5	0.35	1	88.54
CNP0449118	−2	1.25	7.00	2.66	12.63	19.00	13.97	−3.98	−3.77	−3.23	32.97	−1.73	28.88	−3.00	3	−0.39	2	69.67
CNP0348579	−2	1	5.25	4.03	12.14	17.86	9.54	−5.48	−5.24	−4.29	82.57	−1.44	42.46	−2.87	6	0.29	3	84.88
CNP0294912	−1	0	5.25	4.33	12.01	16.94	7.69	−6.05	−5.38	−6.05	1068.85	−0.84	531.63	−2.14	6	0.58	3	100.00
CNP0135500	0	0	6.75	3.50	11.89	17.33	9.06	−4.78	−5.87	−5.62	1056.24	−0.63	1114.00	−2.17	4	0.04	3	100.00
CNP0349353	−1	1	4.75	4.93	11.65	18.28	7.37	−6.34	−5.82	−2.96	137.08	−0.87	299.42	−3.26	3	0.55	1	94.03
CNP0286492	−2	1	6.75	2.77	12.37	18.37	10.47	−4.89	−5.17	−3.73	8.32	−2.67	3.56	−5.06	6	0.01	2	59.66
CNP0290966	1	0	8.75	2.45	12.22	20.36	11.01	−3.33	−4.44	−6.20	249.08	−0.36	121.83	−4.56	6	−0.12	3	84.19
CNP0234840	−2	3	8.40	2.54	12.90	21.13	13.24	−3.85	−4.79	−5.17	239.10	−1.94	105.36	−2.96	8	−0.12	2	84.38
CNP0404801	−2	0	7.50	3.95	14.69	21.09	10.89	−6.07	−6.33	−6.63	251.65	−1.66	111.35	−2.96	6	0.41	3	93.02
CNP0223368	−2	5	7.95	3.30	17.06	28.20	17.66	−5.74	−7.02	−6.41	46.48	−2.87	17.94	−3.96	8	0.36	2	76.09

^a^ CNS: Predicted central nervous system activity on a −2 (inactive) to +2 (active) scale. ^b^ DonorHB: Estimated number of hydrogen bonds that would be donated by the solute to water molecules in an aqueous solution. Values are averages taken over a number of configurations, so they can be non-integer (0.0–6.0). **^c^** AccptHB: Estimated number of hydrogen bonds that would be accepted by the solute from water molecules in an aqueous solution. Values are averages taken over a number of configurations, so they can be non-integer (2.0–20.0). ^d^ QplogPo/w: Predicted octanol/water partition coefficient (−2.0–6.5). ^e^ QPlogPC16: Predicted hexadecane/gas partition coefficient (4.0–18.0). ^f^ QPlogPoct: Predicted octanol/gas partition coefficient (8.0–35.0). ^g^ QplogPw: Predicted water/gas partition coefficient (4.0–45.0). ^h^ QPlogS: Predicted aqueous solubility, log S. S in mol dm^−3^ is the concentration of the solute in a saturated solution that is in equilibrium with the crystalline solid (−6.5–0.5). ^i^ CIQPlogS: Conformation-independent predicted aqueous solubility; log S. S in mol dm^−3^ is the concentration of the solute in a saturated solution that is in equilibrium with the crystalline solid (−6.5–0.5). ^j^ Qplog HERG: Predicted IC_50_ value for blockage of HERG K^+^ channels (concern below-5). ^k^ QPPCaco: Predicted apparent Caco-2 cell permeability in nm/sec. Caco-2 cells are a model for the gut–blood barrier. QikProp predictions are for non-active transport (<25 poor, >500 great). ^l^ QPlogBB: Predicted brain/blood partition coefficient. Note: QikProp predictions are for orally delivered drugs so, for example, dopamine and serotonin are CNS negative because they are too polar to cross the blood–brain barrier (−3.0–1.2). ^m^ QPPMDCK: Predicted apparent MDCK cell permeability in nm/sec. MDCK cells are considered to be a good mimic for the blood–brain barrier. QikProp predictions are for non-active transport (<25 poor, >500 great). ^n^ QPlogKp: Predicted skin permeability, log *K*_p_ (−8.0–−1.0). # metab: Number of likely metabolic reactions. See QikProp descriptor information for a complete list of reactions (1–8). ^o^ Qplog Khsa: Prediction of binding to human serum albumin (−1.5–1.5). ^p^ Human Oral Absorption: Predicted qualitative human oral absorption: 1, 2, or 3 for low, medium, or high. The text version is reported in the output. The assessment uses a knowledge-based set of rules, including checking for suitable values of percent human oral absorption, number of metabolites, number of rotatable bonds, log P, solubility and cell permeability. ^q^ Percent Human Oral Absorption: Predicted human oral absorption on 0 to 100% scale. The prediction is based on a quantitative multiple linear regression model. This property usually correlates well with human oral absorption, as both measure the same property (>80% is high <25% is poor).

**Table 3:** Analysis of hydrogen bond interaction between mIDH1 and the inhibitors.

Complex	Acceptor	Donor	Occupancy (%)	Distance (Å)	Angle (°)
mIDH1-DS-1001b	ligand@O1	ILE_128@N-H	30.30%	3.09	143.57
	ligand@O2	ALA_111@N-H	2.93%	3.06	153.10
	ligand@O2	ILE_128@N-H	2.09%	3.17	145.87
	ligand@O2	ARG_119@NH2-H	1.91%	3.12	131.69
mIDH1-CNP0119040	ligand@O6	LEU_120@N-H	14.08%	3.01	157.39
	ligand@O6	SER_287@OG-H	5.60%	2.76	163.79
	ligand@O1	TRP_124@NE1-H	5.33%	2.94	152.86
	ligand@O6	SER_278@OG-H	4.89%	2.80	159.08
	ligand@O1	ARG_119@NH1-H	1.42%	2.89	152.98
mIDH1-CNP0243438	ligand@O4	TRP_267@NE1-H	2.22%	3.06	139.10
	ligand@O5	TRP_267@NE1-H	1.73%	3.09	138.79
	ligand@O4	ASN_271@ND2-H	1.47%	3.11	153.96
	ligand@O5	TYR_135@OH-H	1.07%	2.93	151.19
	ligand@O4	SER_278@N-H	1.07%	3.19	130.08
	ligand@O1	TRP_267@NE1-H	1.02%	3.22	126.50
mIDH1-CNP0449118	CYS_379@O	ligand@N1-H	16.53%	2.85	141.71
	ligand@O1	SER_287@OG-H	16.44%	2.81	154.95
	ligand@O3	SER_287@OG-H	5.82%	3.23	136.59
	ligand@O2	SER_287@OG-H	4.53%	3.23	145.12

**Table 4:** Calculated binding energy (kcal/mol) between mIDH1 and the inhibitors.

Terms	DS-1001b	CNP0119040	CNP0243438	CNP0449118
ΔE_vdw_	−49.51 ± 3.25	−39.97 ± 3.76	−43.70 ± 6.11	−40.62 ± 2.18
ΔE_ele_	−57.11 ± 7.16	−7.55 ± 3.15	−74.58 ± 16.78	−81.80 ± 8.72
ΔG_gas_	−106.61 ± 8.10	−47.52 ± 4.91	−118.28 ± 20.38	−122.42 ± 8.66
ΔG_GB_	76.29 ± 7.20	24.33 ± 2.97	92.22 ± 16.89	103.23 ± 8.21
ΔG_GBSUR_	−6.63 ± 0.34	−5.55 ± 0.44	−5.26 ± 0.48	−5.56 ± 0.19
ΔG_sol_	69.66 ± 7.12	18.78 ± 2.86	86.96 ± 16.62	97.67 ± 8.20
ΔG_bind_	−36.95 ± 2.96	−28.74 ± 3.93	−31.32 ± 5.99	−24.75 ± 2.24

## Data Availability

PDB, https://www.rcsb.org/, accessed on 28 November 2022; https://coconut.naturalproducts.net/, accessed on 8 November 2022.

## Supplementary Materials

The following supporting information can be downloaded at: https://www.mdpi.com/article/10.3390/ph17030336/s1, Figure S1: The similarity matrix from fingerprint of 12 hits, coumarin and flavonoids; Figure S2: The bioactivity radar and BOILED-Egg plot of the 12 compounds; Figure S3: The Rg values of the DS-1001b, CNP0119040, CNP0243438, and CNP0449118 (frame interval=1); Figure S4: The superposition of RMDS for triplicate sample, ligand(A), pocket(B) and backbone(C); Figure S5: The superposition of RMSF(A) and Rg(B) for triplicate sample; Table S1: The Druglikeness and PAINS of the top 12 compounds.
