# Supplementary material for: Identification of mIDH1 R132C/S280F Inhibitors from Natural Products by Integrated Molecular Docking, Pharmacophore Modeling and Molecular Dynamics Simulations
Source: Pharmaceuticals (Basel). 2024 Mar 5;17(3):336. doi: 10.3390/ph17030336 (PMC10976062; doi:10.3390/ph17030336)
Supplement: Supplementary file 1 [file pharmaceuticals-17-00336-s001.zip › pharmaceuticals-2875108-supplementary.pdf]

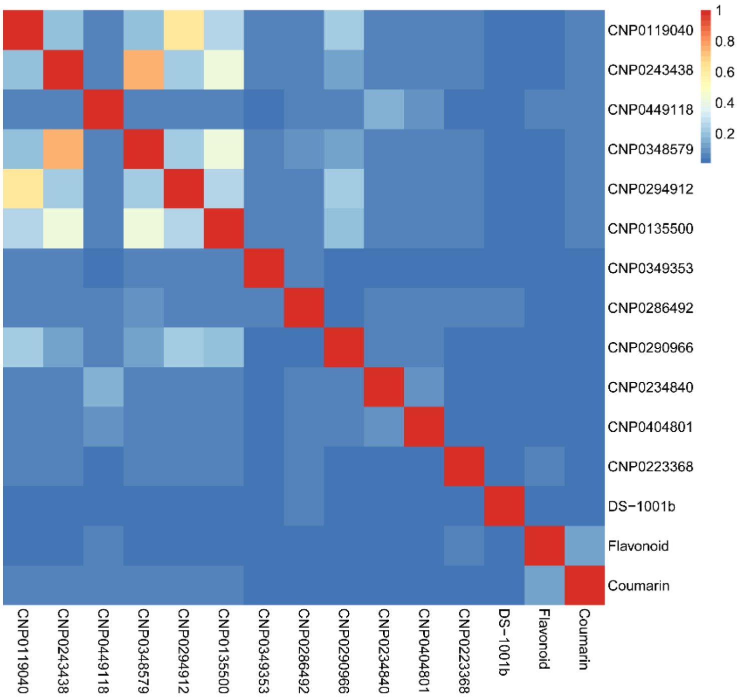

Figure S1. The similarity matrix from fingerprint of 12 hits, coumarin and flavonoids

Table S1. The Druglikeness and PAINS of the top 12 compounds.

ID	Druglikeness						PAINS
	Lipinski	Ghose	Veber	Egan	Muegge	Bioavailability Score	
DS-1001b	No	No	Yes	No	No	0.56	0 alert
CNP0119040	Yes	Yes	Yes	Yes	Yes	0.55	0 alert
CNP0243438	Yes	Yes	Yes	Yes	Yes	0.56	0 alert
CNP0449118	Yes	Yes	Yes	Yes	Yes	0.56	0 alert
CNP0348579	Yes	Yes	Yes	Yes	Yes	0.56	0 alert
CNP0294912	Yes	Yes	Yes	Yes	Yes	0.55	0 alert
CNP0135500	Yes	Yes	Yes	Yes	Yes	0.55	0 alert
CNP0349353	Yes	Yes	Yes	Yes	No	0.85	0 alert
CNP0286492	Yes	Yes	No	No	Yes	0.56	0 alert
CNP0290966	Yes	Yes	Yes	Yes	Yes	0.55	0 alert
CNP0234840	Yes	Yes	No	Yes	Yes	0.55	0 alert
CNP0404801	Yes	Yes	Yes	Yes	Yes	0.55	0 alert
CNP0223368	Yes	No	No	No	Yes	0.55	0 alert

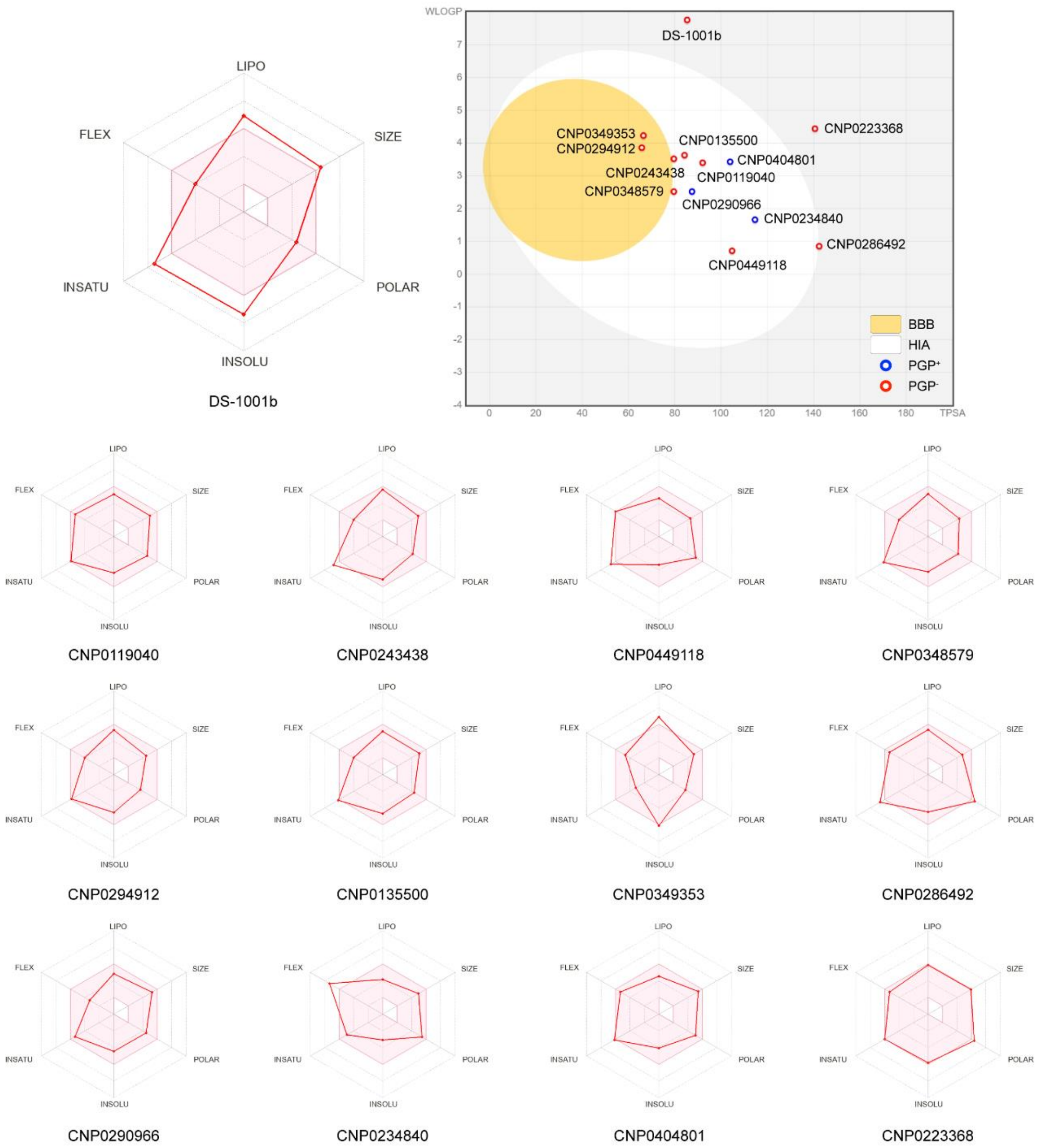

Figure S2. The bioactivity radar and BOILED-Egg plot of the 12 compounds

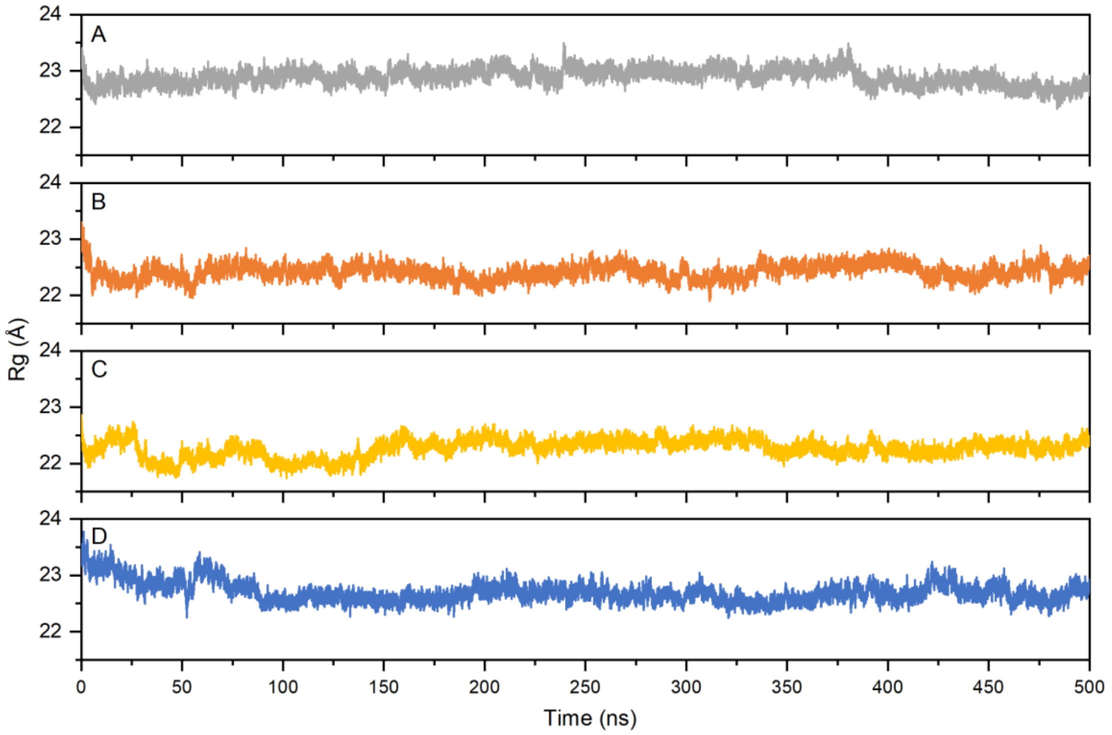

Figure S3. The Rg values of the DS-1001b, CNP0119040, CNP0243438, and CNP0449118 (frame interval=1).

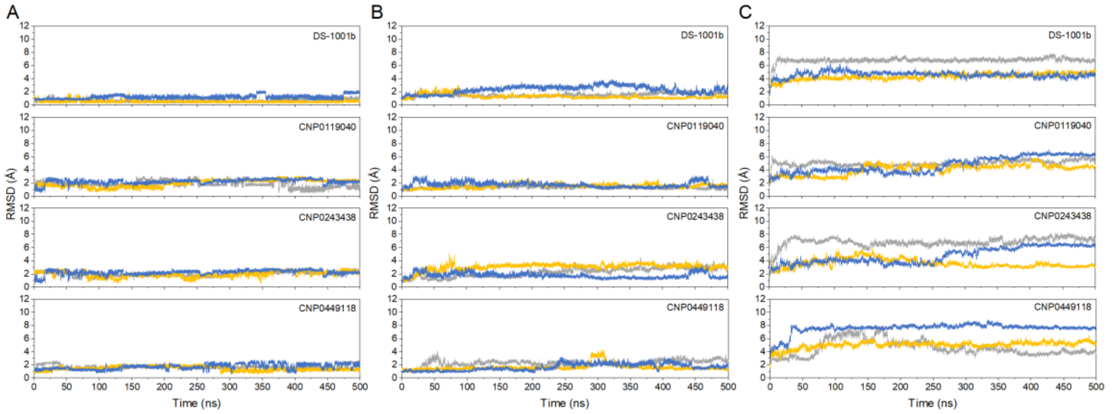

Figure S4. The superposition of RMDS for triplicate sample, ligand(A), pocket(B) and backbone(C).

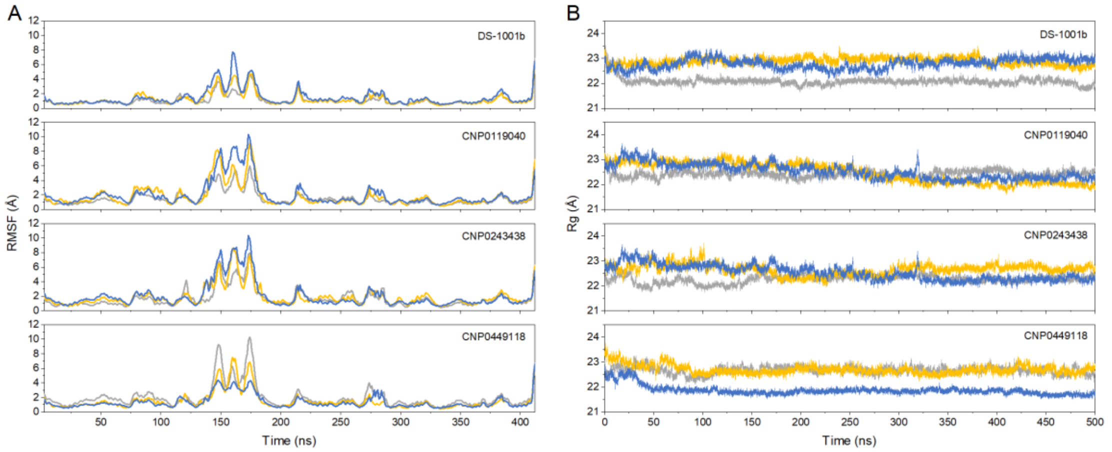

Figure S5. The superposition of RMSF(A) and Rg(B) for triplicate sample.
